# Methodological Approaches to Transform, Develop, and Evaluate Cancer Care Interventions

**DOI:** 10.1093/geroni/igab046.088

**Published:** 2021-12-17

**Authors:** Sean Halpin

## Abstract

Cancer diagnoses in older adults are often abrupt and unexpected, requiring patients to make quick therapy choices. Further, reflecting on traumatic therapy is often difficult. In our symposium, we bring together researchers from varied disciplines to report on patients’ cancer therapy choices. Carrion will discuss her mixed-methods approach to older Latino adults’ cancer therapy preferences. Next, Blackberry will present how patient-derived photographs (photovoice) can improve supportive care for older Australians in facilitating empowerment, patient-centered care, and shared decision making. Halpin, who will apply conversation analysis to examine how multiple myeloma patients interacted with supplemental material during in-person nurse-led education. Last, Seaman will use his work with head and neck cancer survivors to illustrate the conceptual and methodological challenges of investigating those who discontinue care. Understanding how patients from diverse populaces with various cancer diagnoses navigate their therapy may help inform future cancer-related health services’ approaches.

